# High bone mass and cam morphology are independently related to hip osteoarthritis: findings from the High Bone Mass cohort

**DOI:** 10.1186/s12891-022-05603-3

**Published:** 2022-08-06

**Authors:** B. E. Zucker, R. Ebsim, C. Lindner, S. Hardcastle, T. Cootes, J. H. Tobias, M. R. Whitehouse, C. L. Gregson, B. G. Faber, A. E. Hartley

**Affiliations:** 1 Musculoskeletal Research Unit, Translational Health Sciences, Bristol Medical School, University of Bristol, l, Learning and Research Building, Level 1, Southmead Hospita, Bristol, BS10 5NB UK; 2 Division of Informatics, Imaging and Data Sciences, The University of Manchester, Manchester, UK; 3 MRC Integrative Epidemiology Unit (IEU), Bristol Medical School, University of Bristol, Oakfield House, Oakfield Grove, Bristol, BS8 2BN UK; 4 National Institute for Health Research Bristol Biomedical Research Centre, University Hospitals Bristol and Weston NHS Foundation Trust and University of Bristol, Bristol, UK

**Keywords:** Cam morphology, Bone mineral density, Osteoarthritis, High Bone Mass

## Abstract

**Background:**

High bone mass (HBM, BMD Z-score ≥  + 3.2) and cam morphology (bulging of lateral femoral head) are associated with greater odds of prevalent radiographic hip osteoarthritis (rHOA). As cam morphology is itself a manifestation of increased bone deposition around the femoral head, it is conceivable that cam morphology may mediate the relationship between HBM and rHOA. We therefore aimed to determine if individuals with HBM have increased odds of prevalent cam morphology. In addition, we investigated whether the relationship between cam and prevalent and incident osteoarthritis was preserved in a HBM population.

**Methods:**

In the HBM study, a UK based cohort of adults with unexplained HBM and their relatives and spouses (controls), we determined the presence of cam morphology using semi-automatic methods of alpha angle derivation from pelvic radiographs. Associations between HBM status and presence of cam morphology, and between cam morphology and presence of rHOA (or its subphenotypes: osteophytes, joint space narrowing, cysts, and subchondral sclerosis) were determined using multivariable logistic regression, adjusting for age, sex, height, weight, and adolescent physical activity levels. The association between cam at baseline and incidence of rHOA after an average of 8 years was determined. Generalised estimating equations accounted for individual-level clustering.

**Results:**

The study included 352 individuals, of whom 235 (66.7%) were female and 234 (66.5%) had HBM. Included individuals contributed 694 hips, of which 143 had a cam deformity (20.6%). There was no evidence of an association between HBM and cam morphology (OR = 0.97 [95% CI: 0.63–1.51], *p* = 0.90) but a strong relationship was observed between cam morphology and rHOA (OR = 3.96 [2.63–5.98], *p* = 5.46 × 10^–11^) and rHOA subphenotypes joint space narrowing (OR = 3.70 [2.48–5.54], *p* = 1.76 × 10^–10^), subchondral sclerosis (OR = 3.28 [1.60–6.60], *p* = 9.57 × 10^–4^) and osteophytes (OR = 3.01 [1.87–4.87], *p* = 6.37 × 10^–6^). Cam morphology was not associated with incident osteoarthritis (OR = 0.76 [0.16–3.49], *p* = 0.72).

**Conclusions:**

The relationship between cam morphology and rHOA seen in other studies is preserved in a HBM population. This study suggests that the risk of OA conferred by high BMD and by cam morphology are mediated via distinct pathways.

**Supplementary Information:**

The online version contains supplementary material available at 10.1186/s12891-022-05603-3.

## Background

Osteoarthritis (OA) is the most common joint disease, with between 10–12% of the adult population having symptomatic OA [1]. This carries a substantial economic burden, largely attributed to disability and the costs of joint replacement [2]. Hip osteoarthritis (hOA) is one of the most common manifestations of OA with a rising incidence and prevalence globally [3, 4]. The lack of preventative treatments means that increasing emphasis is placed on understanding and mitigating risk factors for its development.

One such risk factor is cam morphology, a bulging of the lateral aspect of the femoral head/neck junction, which has long been associated with hOA, total hip replacement (THR, a proxy for end stage disease) and, more recently, specific subphenotypes of hip OA [5–7]. It has been hypothesized that hip movement, in particular flexion, compresses the acetabular labrum leading to joint degeneration over time [8]. Randomised controlled trials (RCTs) have started to explore surgical options, such as arthroscopic cam resection, as a means of treating pain and possibly preventing progression of OA [9]. Importantly, adolescent activity has been associated with the development of cam morphology and is a potential confounder because it also predisposes individuals to hOA [10–12].

Cam morphology is often defined by the alpha angle (AA), a measure of hip sphericity, which can be derived from 2-dimensional (2D) and 3-dimensional (3D) imaging, with a higher AA used to define cam morphology (most commonly ≥ 60°) [13]. Methods for deriving AA vary but most involve time-consuming manual measurement using different software packages [14–16]. These methods have been found to differ in consistency, with a wide range in inter-rater reliability statistics reported across studies [16, 17]. Recently, semi-automatic methods have been developed with the potential to reduce inter-operator variation, increase reliability and reduce measurement time [18].

High bone mass (HBM), defined as an extreme elevation in bone mineral density (BMD) [19], is a risk factor for OA [20]. HBM individuals have been shown to have increased incidence and progression of radiographic features of OA (*i.e.* osteophytes and joint space narrowing [JSN]) at load bearing joints (knee and hip), as well as an increased prevalence of radiographic OA in non-load-bearing joints (hands) [21–24]. It is thought that these individuals may have a predisposition to a bone-forming phenotype that may be, at least partially, genetically determined [25]. This genetic component may be a result of rare monogenic mutations of large effect or multiple small-effect polygenic variants, or an interaction of the two [26]. Cam morphology itself is a manifestation of increased bone deposition around the femoral head, which may occur after childhood hip diseases or after high levels of physical activity during skeletal development [27–29]. Though cam morphology does not arise through osteophyte or enthesophyte formation, which is increased in HBM individuals [25], to the extent that cam morphology is also more common in this context, this might contribute to the relationship between HBM individuals and hOA.

In this study, we aimed to deploy the latest semi-automatic methods to measure AA on radiographs from the HBM Study and assess the cross-sectional relationships between HBM and cam morphology. In addition, we aimed to assess the relationships between cam morphology and the presence and progression of rHOA and rHOA subphenotypes.

## Methods

### The HBM population

**Fig. 1:**
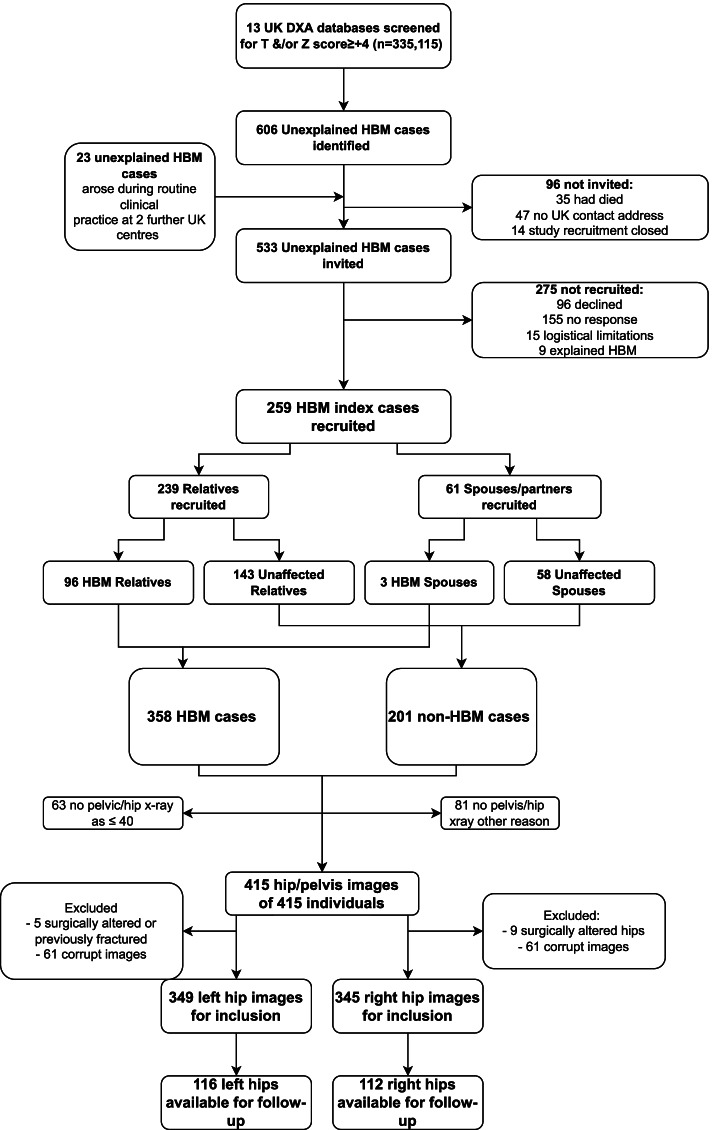
Identification and recruitment of the study population and derivation of the study population. Abbreviations: DXA*:* Dual-energy X-ray absorptiometry, HBM: high bone mass

The study population was derived from the HBM study, a UK based cohort of adults with unexplained HBM. Full details of baseline recruitment, which occurred between 2005–2010, have been published previously [19]. Briefly, index HBM cases were identified by screening routine National Health Service (NHS) DXA databases (335,115 DXA scans from 13 UK DXA databases) for individuals who had *T* and/or *Z*-scores ≥  + 4 at the lumbar spine or hip. All DXA images were screened to exclude scans with artefactually raised BMD (*e.g.* degenerative disease). Though differing in extent, a generalised HBM trait would be expected to affect both spine and hip BMD. L1 Z-score was used as it was not associated with the presence of spinal OA [30]. HBM cases were defined as having a L1 or total hip (TH) BMD *Z*-score ≥  + 3.2 with a *Z*-score ≥  + 1.2 at the other skeletal site. This threshold was consistent with the published precedent for identifying HBM using DXA [31]. Recruited index cases were asked to invite relatives and spouses to undergo DXA screening. Relatives and spouses both with and without HBM were included (Fig. 1). All individuals received assessment including clinical examination and structured interview. Values for age, sex, weight and height were obtained from baseline structured interview and clinical examination. Historical physical activity levels (hours per week of sport between ages 14–21) were determined by postal questionnaire. Informed written consent was obtained from all participants. Participants were re-contacted in 2016 and asked to complete a postal questionnaire and then to attend for follow-up hip radiographs between 2017 and 2018.

### Assessment of bone mineral density

BMD was primarily assessed as continuous variables using maximum (of left and right) TH BMD in g/cm^2^ and L1 BMD in g/cm^2^. These measures were used as the exposures of interest in the relationship between HBM and cam morphology. HBM status (defined above) was used as a binary exposure of interest in the relationship between HBM and cam morphology.

### Assessment of cam morphology

**Fig. 2:**
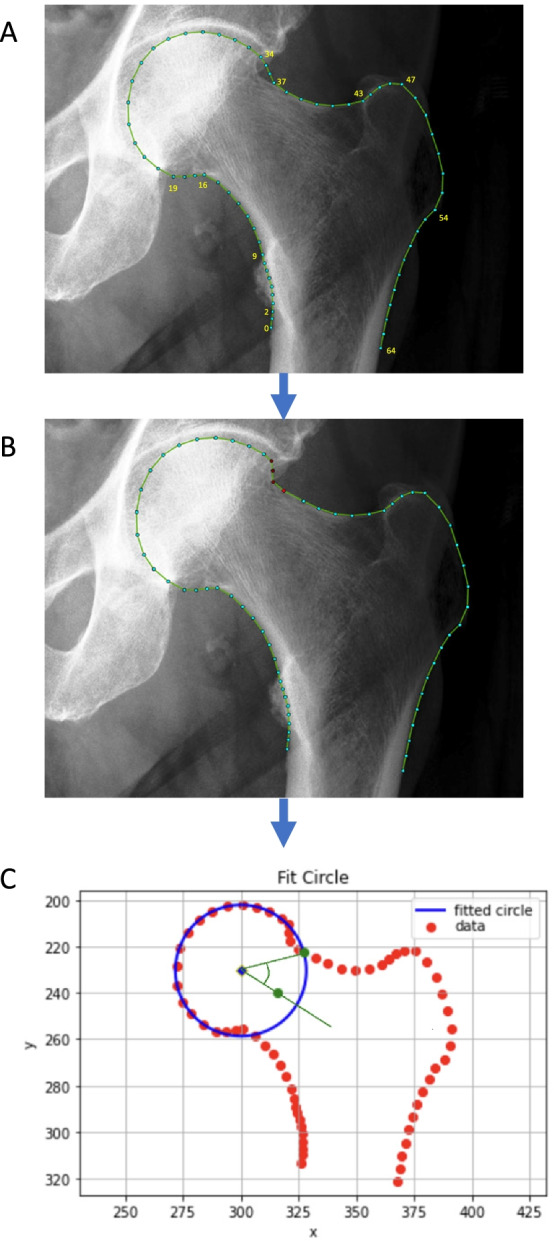
Conversion of plotted points of contour of femoral head and neck in BoneFinder to circle of best fit using custom Python script. Panel **A**: Automated points applied to onto contour of bone using BoneFinder. Anatomically guided points: 0, 2, 9, 16, 19, 34, 37, 43, 47, 55, 65. Panel **B**: Points manually adjusted. Panel **C**: Output of Python script where the red points denote the agreed upon contour of the bone. A circle of best fit around the femoral head is depicted in blue that is fitted to points 19–32. The alpha angle is calculated between a line from the centre point of the circle (blue diamond) to the midpoint of femoral neck (green point, the midpoint of the narrowest section between points 2–15 and 37–49) and a line from the centre of the circle to the location on the femoral head/neck junction where the femur leaves the circle of best fit (green point)

Anteroposterior (AP) pelvic radiographs were performed on the day of structured interview and clinical examination for participants aged over 40 according to local protocols [19]. Both hips were examined. Publicly available BoneFinder software (University of Manchester) was used to automatically place 65 points around the outline of the proximal femur (Fig. 2) [32]. BoneFinder is a machine learning trained algorithm which applies a Random Forest approach to automatically outline the bone with a number of point positions [33]. All points on all images were manually checked and, if necessary, corrected by two raters (BEZ, orthopaedic trainee surgeon and then BGF, rheumatology trainee physician) (Fig. 2). Osteophytes were deliberately excluded as part of the point plotting process. A third rater (MRW, consultant orthopaedic surgeon) was consulted in cases of uncertain optimal point placement (31 hips). Ninety-five left hips had points corrected, 105 right hips had points corrected. The coordinates denoting the agreed upon contour of the bone were then used to determine the alpha angle using a freely available Python 3.0 software package developed on DXA scans (Fig. 2) [18]. Cam morphology was defined as an AA ≥ 60° [13].

We used a random sample of 30 left hips to validate this DXA-derived method on hip radiographs. Blinded AA was derived from the corrected outline points and additionally by using a fully manual method, which has previously been described [18]. Kappas were > 0.80 when comparing the semi-automatic methods to manual gradings of binary cam morphology for both raters. The concordance correlation coefficient (CCC) was 0.72 for rater 1 and 0.98 for rater 2. The Pearson’s coefficient was > 0.85 for both raters. Agreement between raters on cam status was also high (kappa = 0.84), as was agreement on AA derivation (CCC = 0.76, Pearson’s *R* = 0.89).

### Assessment of prevalent radiographic hip osteoarthritis

Methods to assess the study participants for rHOA have been described [20]. Briefly, all available radiographs from both HBM and non-HBM individuals were pooled for assessment to limit observer bias; reasons for unavailability of individual radiographs were ascertained and recorded. Blinded radiographs were graded for Croft score and the presence of radiographic subphenotypes of osteoarthritis including JSN, osteophytes, sclerosis (either femoral or acetabular) and cysts were graded 0–3 using an established atlas [34, 35]. Croft grade ≥ 3 (defined as two of osteophytosis, JSN, subchondral sclerosis or cyst formation) determined the presence of OA. Hips with joint replacements or protheses were recorded and excluded from the main assessment.

### Assessment of incident and/or progressive radiographic hip osteoarthritis

The method of assessment of incident and/or progressive OA has previously been described [22]. Baseline and follow-up pelvic radiographs were pooled for analysis and graded for OA subphenotypes [36], with the reader blinded to HBM status, demographics and timepoint. Endophenotypes were osteophytes, sclerosis, JSN and cysts. OA was graded using Croft score, as described for prevalent OA. Incident OA was defined as the presence of OA in a hip joint that had been free of OA at baseline. Progressive OA was defined as any increase in Croft score in a hip with OA (Croft ≥ 3) at baseline. Progression in osteophyte score and JSN score were defined as any increase in osteophyte score and JSN score between baseline and follow-up.

### Statistical analysis

Demographic statistics for the different study populations were summarised as mean and standard deviation (SD) for continuous variables, and counts and frequencies for categorical variables. The chi-squared test was used to assess the association between binary variables, and the unpaired two-tailed t-test to compare mean values for continuous variables. Analysis of variance was conducted to test for differences in mean AA across investigation centres at which radiographs were taken. Multivariable logistic regression was used to examine associations between BMD variables and cam status, and between cam status and radiographic OA outcome variables. Generalised estimating equations (GEE) using a logistic link function were used to generate an odds ratio accounting for intra-individual clustering between left and right hips. Directed acyclic graphs were used a priori to choose potential confounders for adjustment; analyses were first performed unadjusted (Model 1), then a basic adjusted model included age and sex (Model 2), and then with the covariates weight, height and adolescent physical activity level for a further advanced adjusted model (Model 3). For the analysis of the association between cam morphology and rHOA, we additionally adjusted for HBM status (Model 3). Odds ratios (OR) before and after adjustment for confounders are presented with 95% confidence intervals (95% CI). A sex-stratified analysis was conducted to investigate for sex-differences in the relationship between HBM and cam morphology. A pre-planned sensitivity analysis included testing the association between cam morphology and hip OA defined by a Croft grade ≥ 2. The purpose of this was to increase understanding of the relationship between cam morphology and rHOA. All data were analysed using RStudio (version 1.4.1103).

## Results

### Characteristics of the study population

The HBM study recruited 559 individuals, of whom 352 had satisfactory radiographic data. Reasons for lack of satisfactory radiographic data included: no imaging, imaging files inaccessible, surgically altered hips, or high likelihood of previous hip fracture (Fig. 1). All 352 individuals with satisfactory radiographs were included in the study, 235 (66.7%) were female and 234 (66.5%) had HBM (index cases or relatives with HBM). The mean summed total hip and L1 Z-score in the HBM population was 6.9 (SD 2.1) compared to 1.1 (SD 1.8) in the non-HBM population. HBM individuals were shorter (166.7 cm [SD 8.5] vs. 169.7 cm [9.2]) and heavier (84.7 kg [15.5] vs. 80.8 kg [16.6]) compared to individuals without HBM (supplementary table 1). HBM individuals were slightly older than individuals without HBM (62.5 [11.1] vs. 59.8 [12.9] years). Nearly 20% of all hips had rHOA defined as Croft score ≥ 3 (Table 1). A total of 694 hips were examined for AA and measures of HBM. Of these, 143 were cam hips (20.6%) and the mean AA was 53.9° (15.7). Individuals with a cam deformity were more commonly male (76.4% vs. 39.8%) and were taller and heavier than those without a cam deformity (Table 1). There were no significant differences between mean AA at different investigation centres at which radiographs were taken (*p* = 0.62).

**Table 1:** Demographics of the total study population and prevalence of radiographic hip osteoarthritis features by cam morphology status

	**With Cam**	**Without Cam**	**Total**
	**N (%) of individuals**	**N (%) of individuals**	**N (%) of individuals**
**Female sex**	37 (39.8)	198 (76.4)	235 (66.7)
**Sport aged 14–21**			
0–1 h per week	5 (7.9)	21 (12.1)	26 (11.0)
2–3 h per week	18 (28.6)	51 (29.3)	69 (29.1)
4–7 h per week	15 (23.8)	42 (24.1)	57 (24.0)
Over 7 h per week	25 (39.7)	60 (34.5)	85 (35.9)
**At recruitment**	**Mean (SD)**	**Mean (SD)**	**Mean (SD)**
**Age in years**	62.2 (13.6)	61.4 (11.1)	61.6 (11.8)
**Height in cm**	171.6 (9.0)	166.3 (8.4)	167.7 (8.8)
**Weight in kg**	88.4 (15.5)	81.5 (15.8)	83.4 (16.0)
**Summed total hip and L1 Z-score**	4.7 (3.5)	5.0 (3.3)	4.9 (3.4)
**Max total hip Z-score**	1.2 (0.2)	1.1 (0.2)	1.2 (0.2)
**L1 lumbar spine BMD in g/cm2**	1.3 (0.2)	1.3 (0.2)	1.4 (0.2)
	**N (%) of hips**	**N (%) of hips**	**N (%) of hips**
**OA (Croft > 3)**	57 (40.1)	79 (14.5)	136 (19.8)
**Osteophyte**	119 (83.8)	345 (63.2)	464 (67.4)
**JSN**	60 (42.3)	90 (16.5)	150 (21.8)
**Sclerosis**	15 (10.6)	19 (3.5)	34 (4.9)
**HBM**	81 (56.6)	379 (68.8)	460 (66.3)

*Abbreviations: BMD* Bone mineral density, *OA* Osteoarthritis, *JSN* Joint space narrowing, *HBM* High bone mass, *SD* Standard deviation

### HBM and Cam morphology

Although, a protective association between HBM status and cam status was seen in the unadjusted model (OR 0.59, 95% CI 0.41–0.86, *p* = 6.5 × 10^–3^), this was attenuated by adjustment for sex and age (OR 0.97, 95% CI 0.63–1.51, *p* = 0.90) (Table 2). No association was seen between HBM status and cam status in further sex stratified analysis (Table 3). Furthermore, there was no association between L1 or maximum TH BMD (using continuous measures of BMD) and cam status (Model 3: OR 0.86, 95% CI 0.67–1.09, *p* = 0.21 and OR 0.88, 95% CI 0.68–1.13, *p* = 0.31, OR per SD unit change in BMD).

**Table 2:** Relationships between bone mass and cam morphology, determined by generalised estimating equations

		**Model 1**		**Model 2**		**Model 3**	
**Exposure**	**Outcome**	**OR (95% CI)**	***p*** **value**	**OR (95% CI)**	***p*** **value**	**OR (95% CI)**	***p*** **value**
Max total hip BMD in g/cm^**2**^	Cam	1.12 (0.92–1.36)	0.25	1.09 (0.89–1.33)	0.40	0.88 (0.68–1.13)	0.31
L1 BMD in g/cm^**2**^	Cam	1.03 (0.84–1.25)	0.79	0.98 (0.80–1.20)	0.84	0.86 (0.67–1.09)	0.21
HBM status	Cam	0.59 (0.41–0.86)	6.5 × 10^–3^	0.97 (0.63–1.51)	0.90	0.66 (0.38–1.13)	0.13

Model 1 = unadjusted; Model 2 = age and sex adjusted; Model 3 = adjusted for sex, age, weight, height and adolescent activity

Odds ratios are per SD increase in BMD or the odds in HBM individuals compared to non-HBM individuals

*Abbreviations: HBM* High bone mass, *BMD* Bone mineral density, *OR* Odds ratio, *CI* 95% confidence interval

**Table 3:** Relationships between bone mass and cam morphology, determined by generalised estimating equations, stratified by sex

		**Model 1**		**Model 2**	
		**Male sex**			
**Exposure**	**Outcome**	**OR (95% CI)**	***p*** **value**	**OR (95% CI)**	***p*** **value**
Max total hip BMD in g/cm^2^	Cam	1.11 (0.85–1.47)	0.44	0.90 (0.65–1.25)	0.54
L1 BMD in g/cm^2^	Cam	1.04 (0.80–1.36)	0.75	0.92 (0.65–1.30)	0.62
HBM status	Cam	1.09 (0.65–1.84)	0.74	0.75 (0.38–1.46)	0.40
		**Female sex**			
**Exposure**	**Outcome**	**OR (95% CI)**	***p*** **value**	**OR (95% CI)**	***p*** **value**
Max total hip BMD in g/cm^2^	Cam	1.00 (0.75–1.34)	0.98	0.84 (0.57–1.24)	0.38
L1 BMD in g/cm^2^	Cam	0.92 (0.66–1.28)	0.64	0.74 (0.52–1.05)	0.09
HBM status	Cam	0.87 (0.43–1.74)	0.69	0.55 (0.22–1.37)	0.20

Model 1 = unadjusted; Model 2 = adjusted for age, weight, height, and adolescent activity

Odds ratios are per SD increase in BMD or the odds in HBM individuals compared to non-HBM individuals

*Abbreviations: HBM* High bone mass, *BMD* bone mineral density, *OR* Odds ratio, *CI* 95% confidence interval

### Cam morphology and prevalent radiographic hip OA

Croft score was available for 668 hips (142 hips with cam morphology and 546 without). In unadjusted analyses, there was a strong association between cam morphology and the presence of rHOA (OR 3.96, 95% CI 2.63–5.98, *p* = 5.5 × 10^–11^). Furthermore, cam was associated with radiographic subphenotypes of OA including JSN (OR 3.7, 95% CI 2.48–5.54, *p* = 11.76 × 10^–10^), subchondral sclerosis (OR 3.28, 95% CI 1.62–6.62, *p* = 9.57 × 10^–4^), the presence of osteophytes overall (OR 3.01, 95% CI 1.87–4.87, *p* = 6.37 × 10^–6^), as well as the presence of osteophytes at the acetabulum (OR 2.31, 95% CI 1.50–3.56, *p* = 1.56 × 10^–4^), the lateral femoral head (OR 3.66, 95% CI 2.37–5.66, *p* = 5.01 × 10^–9^) and the infero-medial femoral head (OR 4.49, 95% CI 1.94–10.40, *p* = 4.6 × 10^–4^). The associations between cam morphology and rHOA, JSN and osteophytes persisted after adjustment for age, sex, weight, height, adolescent physical activity level and HBM status (Model 3, Table 4). The sensitivity analysis revealed a strong relationship between the presence of cam morphology and rHOA defined by a Croft score ≥ 2 (OR 3.65, 95% CI 2.45–5.42, *p* = 1.65 × 10^–10^).

**Table 4:** Associations between cam and prevalent radiographic hip osteoarthritis, determined by generalised estimating equations

		**Model 1**		**Model 2**		**Model 3**	
**Exposure**	**Outcome**	**OR (95% CI)**	***p*** **value**	**OR (95% CI)**	***p*** **value**	**OR (95% CI)**	***p*** **value**
Cam	Croft score ≥ 3	3.96 (2.63–5.98)	5.46 × 10^–11^	3.27 (2.10–5.09)	1.60 × 10^–7^	1.95 (1.11–3.43)	0.02
Cam	Osteophyte (any)	3.01 (1.87–4.87)	6.37 × 10^–6^	2.39 (1.41–4.04)	1.21 × 10^–3^	2.31 (1.28–4.20)	5.74 × 10^–3^
Cam	JSN (any)	3.70 (2.48–5.54)	1.76 × 10^–10^	3.19 (2.06–4.96)	2.19 × 10^–7^	2.25 (1.30–3.89)	3.80 × 10^–3^
Cam	Sclerosis	3.28 (1.60–6.60)	9.57 × 10^–4^	2.63 (1.22–5.65)	0.01	1.46 (0.51–4.13)	0.48

Model 1: unadjusted; Model 2: age and sex adjusted; Model 3: adjusted for sex, age, weight and height, adolescent activity level and HBM status

Odds in hips with cam morphology compared to hips without cam morphology

*Abbreviations: JSN* Joint space narrowing, *OR* Odds ratio, *CI* 95% confidence interval

### Cam morphology and incident/progressive radiographic hip OA

Follow-up data for rHOA were only available for 228 hips from 116 individuals, a mean of 8.3 (SD 0.66) years after their first radiograph. The odds of cam hips having rHOA at either baseline or follow-up visit was 2.83 (95% CI 1.00–8.06, *p* = 0.051, Model 3). Incident OA was detected in 17 hips. Cam morphology was not associated with incident OA (Model 3: OR 2.94, 95% CI 0.61–14.27, *p* = 0.18) or any increase in Croft score between baseline and follow-up (Model 1: OR 1.17, 95% CI 0.32–4.27, *p* = 0.81) (Table 5). Furthermore, no evidence was detected for a relationship between cam and change in osteophyte score between baseline and follow-up (Model 1: OR 1.00, 95% CI 0.67–1.49, *p* = 0.99). Similarly, no evidence was detected for a relationship between cam and change in JSN score (Model 1: OR 1.00, 95% CI 0.87–1.15, *p* = 0.99).

**Table 5:** Relationship between cam and incident radiographic OA progression, determined by generalised estimating equations

		**Model 1**		**Model 2**		**Model 3**	
**Exposure**	**Outcome**	**OR (95% CI)**	***p*** **value**	**OR (95% CI)**	***p*** **value**	**OR (95% CI)**	***p*** **value**
Cam	Change in Osteophyte score	1.00 (0.67–1.49)	0.99	1.10 (0.79–1.65)	0.65	1.13 (0.70–1.80)	0.62
Cam	Change in JSN score	1.00 (0.87–1.15)	0.99	0.99 (0.86–1.13)	0.87	1.00 (0.85–1.18)	0.99
Cam	Incident OA	0.76 (0.16–3.49)	0.72	1.28 (0.26–6.37)	0.76	2.94 (0.61–14.27)	0.18
Cam	Increase in Croft score	1.17 (0.32–4.27)	0.81	1.33 (0.34–5.14)	0.68	2.24 (0.48–10.47)	0.31

Incident OA: Any OA at follow-up in a hip with Croft < 3 at baseline

Model 1: unadjusted; Model 2: age and sex adjusted; Model 3: adjusted for sex, age, weight and height, adolescent activity level and HBM status

Odds in hips with cam morphology compared to hips without cam morphology

*Abbreviations: JSN* Joint space narrowing, *OA* Osteoarthritis, *OR* Odds ratio, *CI* 95% confidence interval

## Discussion

We present results from the first observational study to examine the relationship between cam morphology and BMD using a cohort of extreme HBM cases. We found there was no association between BMD, whether measured at the hip or lumbar spine, and the prevalence of cam morphology. Furthermore, there was no association between HBM and the prevalence of cam morphology. We explored the relationship between cam morphology and rHOA in the same population and found that cam morphology was strongly associated with prevalent rHOA. To further understand this relationship, we examined the associations between cam morphology and the subphenotypes of rHOA, which have been investigated less frequently [7]. Similar to the relationship between cam and rHOA, there was a strong association between the presence of cam morphology and the prevalence of osteophytes, JSN and sclerosis, with the strongest associations seen between cam morphology and JSN, and cam morphology and osteophytes, particularly those at the inferomedial femoral head, replicating recent findings from a large DXA based study [7]. The proposed biological mechanism by which cam causes OA describes the bony deformity entering the hip joint on flexion, causing separation of the acetabular cartilage from the labrum [8, 37]. Therefore, one might expect the focus of osteophytes to be around the impinging surfaces, such as the lateral acetabulum and superomedial femoral head. Future research is justified to understand these relationships and their underlying mechanism.

The relationship between cam morphology and rHOA has been observed in a number of studies [5, 38, 39]. However, this is the first study to examine the relationship between cam morphology and BMD. Previous studies have established relationships between HBM and BMD and OA incidence and progression at various joints [40]. In particular, those studies which included this cohort have shown HBM individuals have an increased risk of prevalent hOA (OR 1.52 [CI 1.09, 2.11]) and of progression in OA subphenotypes [20–23]. As cam morphology itself is a manifestation of increased bone deposition around the femoral head it was hypothesised that cam morphology may mediate the relationship between HBM and hOA [27, 29]. However, the results of our study do not support this hypothesis and instead provide evidence that the relationship between cam morphology and rHOA is independent of BMD, acting via separate pathways. There is growing evidence that the HBM phenotype is, at least partially, genetically determined and a result of rare monogenic mutations of large effect and/or multiple small-effect polygenic variants [26]. There is additional evidence that hip shape may be partially genetically determined, and that these genetic influences may overlap with genetic risk factors for osteoarthritis [38]. Whether the genetic influences predisposing to HBM overlap with those influencing hip shape is not yet known. Further research is required to understand these genetic contributions to disease aetiology. In contrast to large population-based studies, our study found no association between the presence of cam at baseline and the subsequent development of rHOA [14, 15, 39]. This finding could be explained by our smaller sample size at follow-up with only 116 individuals (32.9%) having available data. Furthermore, the majority of studies reporting longitudinal associations between cam morphology and incident rHOA have much longer follow-up periods [14, 15, 39].

This is the first study to use novel machine learning based methods to measure AA on radiographs. AA measures were validated against manual methods, and as further validation our results replicate expected relationships in keeping with previously published studies. For example, the male predominance of cam morphology shown by the sex differences in AA are consistent with previous reports [7, 10, 41]. The prevalence of cam identified in our study was slightly higher than previous larger studies [7, 39], although there is a large range in the reported prevalence of cam morphology in the literature with no agreed cam prevalence estimate, largely attributable to the varying AA thresholds and methods used [42]. A large cadaveric study found a prevalence of cam morphology of 26.5%, suggesting our cam prevalence (20.6%) is plausible [43]. This is further substantiated by results from Frank et al., who found a cam prevalence of > 30% in asymptomatic individuals [44]. In terms of our findings between cam morphology and rHOA these are also consistent with results from previous studies which report ORs 1.05—3.67 [14, 39].

### Strengths and limitations

This study is the first to investigate the relationship between BMD and cam morphology, and between cam morphology and rHOA, in a population of individuals with extreme elevations of BMD [19]. Another strength of this study is our use of novel semi-automatic methods of deriving AA which increase the inter-rater reliability of derivation and improve comparisons between studies. However, a limitation of our study stemmed from the multi-centre nature of recruitment. Positioning is thought to be important when investigating hip radiographs [45], as such the lack of a standardised positioning protocol across centres may predispose to high inter-centre variability in AA derivation. Despite this, no meaningful differences in AA were noted across sites. Furthermore, AP radiographs may be limited in their ability to detect anterior cam lesions compared to other 2-D views, 3-D imaging or cadaveric studies [10, 43, 46]. This may have resulted in under-identification of cam morphology in this study. However, it is unlikely that this measurement error was differential between those with and without hip OA; thus any bias would be towards the null. As similar associations between cam morphology and hOA were seen in previous studies, this suggests our methods were sufficient to assess cam morphology on a population level [5, 38, 39].

The study population suffered from a relatively high attrition rate between baseline and follow-up resulting in reduced statistical power in the detection of OA incidence and progression. This is largely due to the older age of the population at recruitment meaning follow-up was limited by death and poor health. This likely resulted in a follow-up population in which individuals were less likely to have significant OA, therefore introducing bias towards the null. Finally, the study of a population comprising individuals with extreme elevations of BMD limits the generalisability of these results. However, the use of this population is integral to the understanding of the mechanisms by which BMD influences OA. Further research is required to understand whether BMD is associated with cam morphology in less extreme populations.

## Conclusions

This observational study, using novel methods of alpha angle derivation applied to plain hip radiographs, identified a strong relationship between cam morphology and prevalent rHOA in a population comprising individuals with extreme elevations in BMD. This study found no relationship between cam morphology and BMD, lending credence to our new hypothesis that their individual relationships with rHOA are mediated by distinct pathways. Further work is needed to understand whether the absence of a relationship between cam and BMD is replicated in individuals with less extreme elevations of BMD.

## Supplementary Information

**Additional file 1: Supplementary table 1. **Demographics of the total study population by high bone mass status.

## Data Availability

The data used in this study is available for all researchers. Please contact the corresponding author to arrange access.

## Abbreviations

2D: 2-Dimensional
3D: 3-Dimensional
AA: Alpha Angle
AP: Anteroposterior
BMD: Bone mineral density
CI: 95% Confidence interval
CCC: Concordance correlation coefficient
GEE: Generalised estimating equations
HBM: High bone mass
hOA: Hip osteoarthritis
JSN: Joint space narrowing
NHS: National Health Service
OR: Odds ratios
RCTs: Randomised contolled trials
SD: Standard deviation
THR: Total hip replacement
