# Supplementary material for: High bone mass and cam morphology are independently related to hip osteoarthritis: findings from the High Bone Mass cohort
Source: BMC Musculoskelet Disord. 2022 Aug 6;23:757. doi: 10.1186/s12891-022-05603-3 (PMC9356486; doi:10.1186/s12891-022-05603-3)
Supplement: Supplementary file 1 — Additional file 1: Supplementary table 1. Demographics of the total study population by high bone mass status. [file 12891_2022_5603_MOESM1_ESM.docx]

**Supplementary table 1. Demographics of the total study population by high bone mass status**

|  | With HBM | Without HBM | Total (%) |
| --- | --- | --- | --- |
|  | **N (%) of individuals** | **N (%) of individuals** | **N (%) of individuals** |
| Female sex | 177 (75.6) | 58 (49.2) | 235 (66.7) |
| Participation in sport aged 14-21 years |  |  |  |
| 0-1 hour per week | 17 (10.8) | 9 (11.4) | 26 (11.0) |
| 2-3 hrs per week | 46 (29.1) | 23 (29.1) | 69 (29.1) |
| 4-7 hrs per week | 38 (24.1) | 19 (24.1) | 57 (24.0) |
| > 7 hrs per week | 57 (36.1) | 28 (35.4) | 85 (35.9) |
|  | **Mean (SD)** | **Mean (SD)** | **Mean (SD)** |
| Age at recruitment, years | 62.5 (11.1) | 59.8 (12.9) | 61.6 (11.8) |
| Height at recruitment, cm | 166.7 (8.5) | 169.7 (9.2) | 167.7 (8.8) |
| Weight at recruitment, kg | 84.7 (15.5) | 80.8 (16.6) | 83.4 (16.0) |
| Summed total hip and L1 Z-score | 6.9 (2.1) | 1.1 (1.8) | 4.9 (3.4) |
| Max total hip BMD in g/cm^2^ | 1.3 (0.2) | 1 (0.1) | 1.2 (0.2) |
| L1 BMD in g/cm^2^ | 1.4 (0.2) | 1.1 (0.2) | 1.4 (0.2) |

*Abbreviations: HBM: high bone mass, SD: Standard deviation, BMD: bone mineral density*
